# Harvesting cereals at Tappeh Sang-e Chakhmaq and the introduction of farming in Northeastern Iran during the Neolithic

**DOI:** 10.1371/journal.pone.0290537

**Published:** 2023-08-25

**Authors:** Fiona Pichon, Juan José Ibáñez Estevez, Patricia C. Anderson, Akira Tsuneki

**Affiliations:** 1 Archaeology of Social Dynamics (ASD), Institución Milá y Fontanals (IMF), Spanish National Research Council (CSIC), Barcelona, Spain; 2 Archéorient—Environnements et Sociétés de l’Orient Ancien, UMR 5133, CNRS, Lyon, France; 3 CEPAM—Culture et Environnements, Préhistoire, Antiquité, Moyen-Age, UMR 7264, CNRS, Nice, France; 4 Faculty of Humanities and Social Science, University of Tsukuba, Tsukuba, Japan; New York State Museum, UNITED STATES

## Abstract

Tappeh Sang-e Chakhmaq is the only Neolithic site in Northeastern Iran, characterised by aceramic and ceramic levels corresponding to an occupation of 1500 years from the eighth to the end of the sixth millennium BCE. The Western and Eastern Mounds represent the oldest and longest occupation among the sites identified East of the Zagros, providing a unique context to explore the origin and spread of farming outside the core area of the Eastern Fertile Crescent. We present data about the first harvesting activities in the Northeastern Iranian Central Plateau by applying usewear and microtexture analysis through confocal microscopy on sickle gloss blades. Our results indicate a community of pioneer farmers who settled down in the area carrying with them both domestic cereals as well as advanced techniques of cereal cultivation. We demonstrate that most of the tools were used for harvesting cereals in a fully ripened state collected near the ground, indicating a well-established cereal cultivation strategy. The use of straight shafts with parallel inserts in Tappeh Sang-e Chakhmaq, as known in some sites in the Zagros, suggests the dispersal of farming practices and technologies from the Eastern Fertile Crescent north-eastward across Iran. We observe an evolution in the degree of ripeness of harvested cereals along the first four levels of occupation of the Western Mound, where semi-ripe harvesting is relatively important, suggesting that domestic cereals to be harvested before full maturity were introduced into the village. From the topmost of the Western Mound and along the occupation of the Eastern Mound, ripe harvesting is dominant, showing a well-established cultivation strategy of fully mature cereal. This shift could indicate an *in-situ* evolution towards a better-established agricultural technology, including harvesting riper crops, that would have resulted in higher yields, as cereals were collected when the grain was fully formed.

## Introduction

The slow transition from hunting and gathering of wild animals and plants to crop cultivation and animal domestication, and later to agriculture based on farming, is one of the most critical transformations in human history that occurred independently in many areas of the world [1, 2]. In Southwest Asia, throughout several millennia, from the Late Pleistocene to the Early Holocene (ca. 13,000–7000 BCE), human communities turned from mobile hunter-gatherers to settled farmer-herders [3–8], a shift leading to dramatic changes in their lifeways and in their relationship with the surrounding environment [9–14]. Studies on the emergence of agricultural communities in Southwest Asia emphasize the polycentric and protracted nature of the changes, the various causes − i.e., climatic, ecological, demographic, and symbolic − involved in the process, and hence the multiple regional pathways of the Neolithic transition across the Fertile Crescent ([15–28]. During the 1950s-70s, and following Braidwoods’ pioneering interdisciplinary fieldwork in the Central Zagros [29–31], numerous archaeological projects have revealed the significance of the transition to agriculture across Southwest Asia. The spread of cultivation and farming in the Eastern Fertile Crescent (eastern Iraq to western/southern Iran) had been the focus of much of this research [32–41]. In the late 1970s in Iran and 1980s in Iraq, however, investigations were severely affected by unstable political conditions [42], and research focused primarily in the Levant and southeast Anatolia, resulting in an emphasis on the role of these regions for the origin of the Neolithic in Southwest Asia [43, 44]. Systematic fieldwork resumed during the 1990s, particularly along the Zagros hilly flanks, raising once again attention on the relevant role of the area during the early stages of the Neolithic [45–57]. Recently, new archaeological evidence indicates an early Neolithic occupation, dating back to 10,000/9,500 years BCE, of the Central Zagros area [54, 58–60]. Indeed, evidence of human management of wild and domesticated animals [61–64] and legume founder crops [65] support the idea of the early development of farming and settled communities in the Zagros-Taurus arc, with domestic-type cereals (emmer) appearing for the first time in the area around 7,800 BCE [21, 66]. By contrast, the origin of Neolithic lifeways and agriculture in the area East of the Zagros is still poorly investigated [42, 60, 67, 68]. The eastern part of the Iranian plateau has to a large extent been unexplored [69, 70], and evidence for Early Holocene settlements is scarce [71], raising challenging questions about the mechanisms of the Neolithisation process, including plant and animal management practices, early domestication, and adoption of new technologies, following the last Ice Age [67–69, 72–76]. Moreover, the recurrent stratigraphic hiatus between the occupations related to the foraging Epipalaeolithic and later Neolithic, together with the lack of evidence of wild fauna and plant resource management preceding the exploitation of fully domesticated species, does not support an *in situ* origin hypothesis of an agricultural economy [76, 77].

Among the Neolithic material culture, harvesting technology can provide essential clues about the conditions in which agriculture was transmitted and practised. Techno-typological and functional analyses of harvesting tools also provide crucial information about agricultural technical traditions (i.e., cereals management, collection, and post-harvest processing) and Neolithic farming systems development. For example, the geographical distribution of different types of sickle inserts, combined with radiocarbon dates, was used to reconstruct the place of the Neolithic expansion and the spread of technological innovations westwards in the Mediterranean basin [78, 79]. The technological investment in the manufacturing of the sickles, including the production and rejuvenation of the stone inserts, varies in time and space along with the increasing reliance on domestic cereals crops, reflecting regional cultural dynamics and innovative adaptation solutions [80–89].

**Fig 1:**
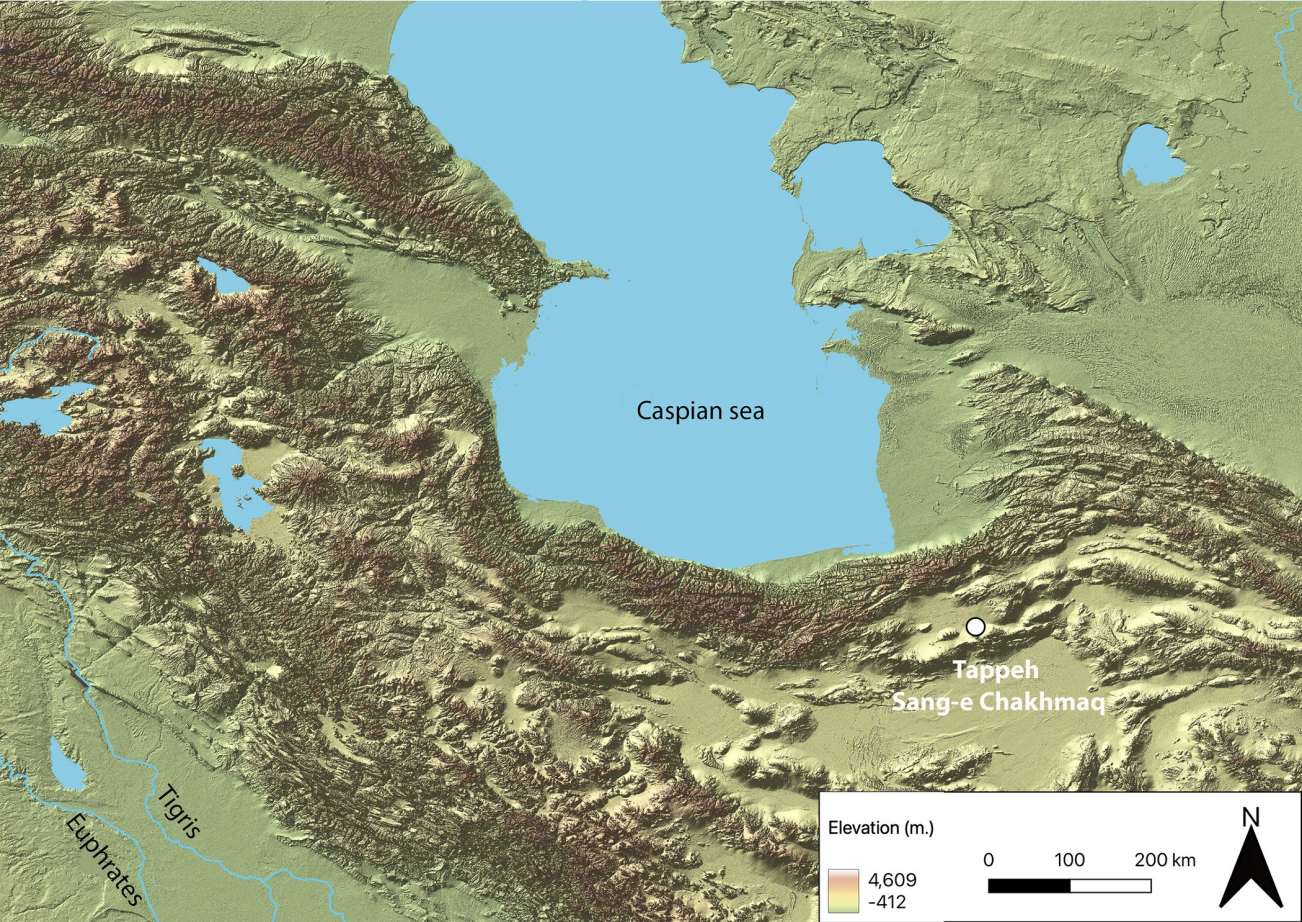
Location of the Neolithic site of Tappeh Sang-e Chakhmaq in the Northeastern Iranian Central Plateau (source map: USGS National Map Viewer).

Through the techno-functional analysis of sickles coming from the Neolithic site of Tappeh Sang-e Chakhmaq (Fig 1), we present data about harvesting activities and the expansion of cereal agriculture in the Northeastern Iranian Central Plateau. Tappeh Sang-e Chakhmaq has an early and long occupation sequence, ca. 1500 years, from the eighth to the late sixth millennium BCE [74, 90, 91]. To date, the extensive excavations carried out in the 1970s by the Japanese archaeological team from the Tokyo University of Education provided the most comprehensive dataset for studying the establishment and spread of farming life in the region [90, 92]. Furthermore, Tappeh Sang-e Chakhmaq is also one of the only known Neolithic sites in the area with reliable evidence of carpological remains [74, 93] and represents a unique opportunity to explore the current research question about the harvesting technology in an early agro-pastoral community outside the "core area" of the Eastern Fertile Crescent.

This paper presents the results of the techno-typological and functional analysis of glossy flint blades coming from both the West and East mounds of Tappeh Sang-e Chakhmaq. Our work focuses on experimental replication, microwear analysis, and on the first application of confocal microscopy and surface texture analysis on Neolithic harvesting tools from the eastern Fertile Crescent context. In our study, we 1) interpret harvesting activities by collecting evidence for reaping when cereals were partially green or completely ripe and evidence for low or high cutting of the crops; 2) describe the morphology of the sickle inserts, and 3) examine evidence for change or continuity in the sickle insert production and use within the occupation sequence of both mounds at Tappeh Sang-e Chakhmaq. The evidence for harvesting practices is compared with data from other regions in our examination of the beginning of cereal agriculture in the Northeastern Iranian Central Plateau.

## Tappeh Sang-e Chakhmaq

Tappeh Sang-e Chakhmaq ("Flintstone Mound" in Persian) is located on the plain of Bastam on the northern edge of the Iranian Central Plateau (ICP), between the Alborz Mountains and the Central Desert (Dasht-e Kavir), 8 km north of the town of Shahroud [74, 90, 92, 94–97]. The site is situated near the edge of one alluvial fan, part of the southeastern foothills of the Alborz Mountains, approximately 1400 m above sea level [70]. Its placement within a Mediterranean-type dry-summer climate zone characterised by fertile soils and numerous water springs that emerged from the edge of the alluvial fan represents an ideal environment for settlement, offering valuable and attractive resources for the early farmers practising dry cereal cultivation [67, 92].

Discovered and first excavated during four seasons in the 1970s by a team led by Seiichi Masuda of Tokyo University of Education (in 1971, 1973, 1975, and 1977), the site comprises two adjacent mounds, the West Mound and the East Mound, located on the same plateau some 200 m apart [90]. AMS dating places the occupation of the West Mound between 7,200 and 6,600 years cal BCE [91]. The occupation of the East Mound took place after a chronological gap of several hundred years, around 6,300 cal BCE [91]. Newly obtained radiocarbon dates confirm that the West Mound of Tappeh Sang-e Chakhmaq was occupied from 7,000 cal BCE until 6,700 cal BCE, while the East Mound was first inhabited ca. 6,200–6,100 cal BCE until around 5,300 BCE [74]. In 2009, K. Roustaei from the Iranian Center for Archaeological Research conducted a short exvacation on the two mounds obtaining new data about the Neolithic subsistence practices through a systematic examination of fauna and flora remains [74, 97].

### The West Mound (or Western Tappeh-WT)

According to the Japanese fieldwork, the West Mound of Tappeh Sang-e Chakhmaq is divided into five occupation layers (I-V from top to bottom) [90]. The erosion of the uppermost level (level I) did not allow the retrieval of any architecture. Levels II to V are associated with the Pre-Pottery Neolithic. Here, rectangular mudbrick houses characterised by one-room and with plastered red floors are present. The chipped stone industry is made on both local flints (light brown and grey flint and chalcedony) and, to a lesser extent, obsidian (6–7% of the whole chipped stone assemblage) [98]. The blades and bladelets are produced by pressure flaking from single-platform cores, which could reach up to 20 cm in length [90]. Tools comprise sickle blades, laterally retouched or truncated bladelets and blades, drills, geometrics, and end-scrapers [74, 90]. Laser Ablation Inductively Coupled Plasma Mass Spectrometry performed on seven obsidian flakes from the recent excavations indicate their origin from Bingöl B and Bingöl A (respectively Alatepe’s and Solhan’s outcrops), West of Lake Van in Turkey [68]. Tappeh Sang-e Chakhmaq West Mound is the easternmost site in northern Iran where Anatolian obsidian has been identified.

The analyses of the archaeobotanical remains coming from the recent excavations showed that the subsistence economy included cereal cultivation–presence of domesticated wheat and barley–from the earliest levels of the West Mound [74, 99]. One and two-grained einkorn (T. monococcum) and a tetraploid hulled wheat (T. turgidum or T. timophevii) are the main crops remains. Diverse wild plants, including grasses (Aegilops, Bromus, Eremopyrum, Poaceae), sedges (Cyperaceae), as well as wild pulses (Astragalus), are gathered too. The subsistence economy was also based on the herding of goats and the hunting of wild sheep, goats, and gazelle [74].

### The East Mound (or Eastern Tappeh-ET)

Based on architectural features unearthed during the first excavations, the East Mound is characterised by six ceramic Neolithic occupation levels (I-VI, from top to bottom) [90]. Here, cigar-shaped mudbrick and rectangular-plan buildings, with large circular and ovoid hearths and kilns, are present [90, 92, 98]. The red-on-buff painted pottery with geometric patterns is attested throughout the sequence [100]. Another type of painted pottery, appearing in layer III and becoming dominant in layers II and I, is characterised by a reddish-brown exterior surface decorated with black painted geometric and naturalistic patterns, showing some similar attributes are with Sialk II/Cheshmeh Ali type pottery (dating to the 6^th^ millennium) from central Iran [100]. In comparison with the West Mound, little changes are recorded in the chipped stone assemblage. Lithic raw materials are always light-brown and grey flints. Blades and bladelets are detached from unilateral-platform cores through pressure flaking [90]. As for the West Mound, the most common tools are sickle elements, geometrics, drills, and end-scrapers. Obsidian artefacts are rare (ca. 1% of the lithic assemblage) [92]. However, new raw materials are found in the lithic assemblages, including turquoise, marble, alabaster, and shale [74].

The analyses of the archaeobotanical remains coming from the recent excavations showed that the hulled tetraploid wheat (T. turgidum or T. timopheevii) is still dominant amongst the identified taxa (Roustaei et al. 2015). The exploitation of a free-threshing wheat (T. aestivum) is attested, while einkorn is no longer represented, suggesting a change in the crop assemblage through time [99]. As shown at the West Mound, barley is poorly represented. Finally, the proportion of wild plants is decreasing considerably. Domesticated small ruminants, goats, and sheep, are dominant, and cattles are yet incorporated into the domestic package [74]. Hunting of gazelle, red deer, and onager are attested.

## Material and methods

### Inclusivity in global research

The cultural material studied in this paper is stored in the Research Center for West Asian Civilization, Department of History and Anthropology, University of Tsukuba (Tsukuba, Japan). The material was collected during the four seasons of excavations led by Seiichi Masuda of the Tokyo University of Education in the 1970s. Permission for these excavations was given to Prof. Sei-ichi Masuda from the Iranian Center for Archaeological Research in 1971. The excavated materials were divided between ICAR and Prof. Sei-ichi Masuda (from Tokyo University of Education) based on the cultural heritage protection law of Iran, and some of the divided materials were exported to Japan in 1975. Additional information regarding the ethical, cultural, and scientific considerations specific to inclusivity in global research is included in the Supporting Information (S1 Checklist).

### Techno-functional analysis of sickle blades and usewear methods

Macrowear and microwear analysis of sickle blades was conducted to collect specific data about ancient harvesting methods. The intensity of sickle use, modes of uses, and hafting techniques were documented. Experimental studies have demonstrated how cutting siliceous plants (e.g., cereals, various types of grass, and reeds) produces characteristic usewear traces on the flint tools, namely a microwear gloss along both faces of the active edge [101]. The selection of glossy tools was carried out using a Perfex Science binocular magnifier (up to 45x magnification). At these lower magnifications, all types of glossy surfaces, even the most marginal ones, can be identified. Before proceeding with the analyses, the archaeological tools– and experimental ones used as a reference collection –were cleaned with soapy water. The 106 blades sampled for the West Mound and the 100 blades for the East Mound were then analysed at higher magnification with an Olympus BHM reflected-light optical microscope (100x, 200x, 400x) to identify the texture, and the types of micropolish and striations [102, 103]. Following the *chaîne opératoire* approach, various technological and typological features were also analysed and recorded, including type of raw material, blank type and shape, butt type, pattern of knapping, position and type of retouch, and formal tool dimensions.

### Quantitative texture gloss analysis through confocal scanning microscopy

Quantitative approaches are common in functional analyses of lithic artifacts. Confocal microscopy and texture analysis have been employed in recent studies of microwear polishes [104–106]. Through the texture analysis of sickle gloss on experimental and ancient stone tools, a method of identifying the types of plants that were harvested in ancient times has been developed [27, 107, 108]. This method was employed in studies of harvesting tools from Epipaleolithic and Early Neolithic Levantine contexts. Variations in the gloss that formed on experimental sickles used to cut ripe domesticated cereals, semi-ripe cultivated wild cereals, green cereals reaped in natural stands, reeds, and grasses were documented and compared with patterns of sickle gloss of ancient tools, documenting the progressive cutting of riper cereals during the origins of cereal agriculture [107, 108]. In this study, the same methods are used to compare archaeological sickle gloss with patterns observed on experimental tools used to cut domestic ripe cereals, semi-ripe cultivated wild cereals, reeds, and grasses. We have not included cutting wild cereals in natural stands in the experimental reference collection, as we did in previous studies analysing harvesting practices in the Levant, as the Northeastern Iranian Central Plateau was not a region in which wild cereals grew spontaneously [15]. The aim of this study is to know whether glossed tools at Tappeh Sang-e Chakhmaq were used to harvest cereals, reeds, or grasses. Moreover, for those tools identified as sickle elements used to cut cereals, we want to identify whether the cereals were reaped in full maturity or cut in a semi-ripe state. This question is important, as it has been observed that the origins of cereal agriculture are associated with the transition from green to ripe cutting of cereals [27]. We also applied confocal microscopy and texture analysis of harvesting polish to document different patterns on experimental tools used to cut cereals at different heights (e.g., near the ground, higher up near the spikelets, etc.) [109]. Low cutting of the crops makes easier the transport of the yield in bundles to the threshing areas and allows the use of the straw for different activities (livestock feeding, basketry, roofing), but makes the threshing activities more time-consuming than high cutting the crops.

Our methodological approach has been described in detail in previous papers [27, 104, 107, 109]. All the sampled archaeological tools showed well-developed macropolish, visible with the naked eyes. For each experimental and archaeological tool, 3 to 14 areas of 650×500 μm were measured along the edge. Measurements were made across the portions of the edge where the micropolish is best developed (i.e., 90% of the sampled surface) using Sensofar Plu Neox scanning confocal microscope, with a 20X (0.45 NA) objective, with a spatial sampling of 0.83 mm, an optical resolution of 0.31 μm, a vertical resolution of 20 nm and a z-step interval of 1 μm. Confocal microscopes provide very precise measures of microtextures, but a small amount of system noise exists, measured as the difference between two consecutive measures of the same surface. To reduce it, it is convenient to threshold the surface measures, eliminating a certain proportion of the measured surface. Thus, 99.5% of the surface was scanned, while the rest of the area (0.05%) was not considered, and the non-measured points were later filled. The 3D images generated by the confocal microscope were processed using the Mountain 7 software from Digital Surf to extract ISO 25178 parameters of texture measurement. Subsamples of 200×200 μm, with homogeneous and well-developed micropolish without irregularities caused by the natural surface of the flint, were taken from the 650×500 μm large areas resulting in a minimum of 15 subareas for each tool. Several leveling operators and spatial filtering available in the software were used to correct the horizontality of the surface and isolate the roughness component of the micropolish from the source surface. The statistical analysis has been performed with SPSS, building predictive models for archaeological glossed tools through the quadratic discriminant analysis of experimental groups.

### Discriminating between harvested plant types

To discriminate between the types of harvested plants, 14 experimental tools used to cut semi-ripe cultivated wild cereals, ripe domestic cereals, reeds, and grasses were selected as a reference framework that was compared with the wear traces on the archaeological tools (Table 1). For each experimental tool, the number of measured areas (650×500 μm) varied from 18 to 35, for a total of 316 of 200×200 μm subareas.

**Table 1:** The experimental sickles used to harvest different types of plants.

Tool n°	Type of plants	Plant species	Year	Place	Time of use	Sickles
**1**	Domestic cereal	*T*. *spelta*	1994	Zureda, Asturias (Spain)	420’	One sickle
**2**	Domestic cereal	*T*. *spelta*	1994	Zureda, Asturias (Spain)	420’	
**3**	Domestic cereal	*T*. *spelta*	1994	Zureda, Asturias (Spain)	420’	
**4**	Domestic cereal	*T*. *spelta*	1994	Zureda, Asturias (Spain)	270’	One sickle
**5**	Domestic cereal	*T*. *monoccocum*	2008	Seranon, Alpes-Maritimes (France)	270’	One sickle
**6**	Domestic cereal	*T*. *aestivum*, *T*. *monococcum*, *T*. *dicoccum*	1997	Jalès, Ardèche (France)	900’	One sickle
**7**	Wild cultivated	*T*. *boeticum*	1992	Jalès (France)	480’	One sickle
**8**	Wild cultivated	*T*. *boeticum*	1992	Jalès (France)	600’	One sickle
**9**	Wild cultivated	*T*. *boeticum*	1989	Jalès (France)	480’	One sickle
**10**	Wild cultivated	*T*. *boeticum*	1989	Jalès (France)	760’	One sickle
**11**	Wild cultivated	*T*. *boeticum*	1989	Jalès (France)	480’	One sickle
**12**	Reeds	*Phragmites communis*	1993	Jalès (France)	100’	One sickle
**13**	Reeds	*Phragmites communis*	1993	Jalès (France)	120’	One sickle
**14**	Grass	*Ampelodesmos mauritanica*	2012	Ain Salem (Tunisia)	80’	One sickle

Eleven ISO 25178 surface parameters were selected based on their capacity to discriminate the four experimental groups through quadratic discriminant function analysis [107]. The parameters include: 1) *Amplitude parameters*, a class of surface finish parameters characterising the distribution of heights (Sa, the mean height of the surface; Sq, the square root mean height; Sz, the distance between the highest peak and the deepest valley; Sp, the maximum peak height and Sv, maximum valley depth area); 2) *Spatial parameters*, which quantify the lateral information present on the X and Y-axes of the surface based upon spectral analysis (Sal, expressing the content in wavelength of the surface; Str, which measures whether the surface is isotropic); 3) *Hybrid parameters* considering both the amplitude and the spacing (Sdq, the root-mean-square value of the surface slope; Sfd, indicating the complexity of the surface using the fractal dimension theory); 4) *Parameters measuring the micro-valley network*, obtained after the vectorisation of the surface, searching for all the furrows in a surface and measuring their mean depth (MDF) and mean density (MDenF).

Quadratic discriminant function analysis builds a predictive model for group membership, which is composed of discriminant functions based on quadratic combinations of predictor variables when these variables show different variance-covariance matrices. The classification rule of the predictive analysis is based on Bayes’ theorem. More than 70% of the 3D surfaces were correctly grouped using quadratic discriminant analysis (Table 2).

**Table 2:** Predicted group membership through quadratic discriminant classification of the experimental samples; 76.2% of original grouped cases were correctly classified.

Predicted group membership										
	Domestic ripe cereal		Wild cultivated semi-ripe cereal		Reeds		Grass		Total	
	N	%	N	%	N	%	N	%	N	%
**Domestic ripe cereal**	118	78.1	28	18.5	4	2.6	1	0.7	151	100
**Wild cultivated semi-ripe cereal**	24	22.9	74	70.5	6	5.7	1	1.0	105	100
**Reeds**	4	9.1	6	13.6	33	75.0	1	2.3	44	100
**Grass**	0	0	0	0	0	0	15	100	15	100

Significant mean differences (Wilks’ Lambda <0,001) were observed for all the predictors mentioned in the previous section (except for Sfd, which is 0,016) and for the three discriminant functions. While the log determinants were quite similar, Box’s M indicated that the assumption of the equality of covariance matrices was violated, so a quadratic discriminant analysis was chosen.

To test the capacity for correct classification of our predictive model more clearly, we predicted the group membership of the fourteen experimental tools (Table 3). All of them were correctly classified, with more than 55% of 3D images correctly grouped, except tool 10, a flint blade used to harvest cultivated wild cereals, for which correct classification only reaches 41,7% of the 3D images. As less than 55% of the images were not classified in any experimental group, if this tool had been an archaeological one (corresponding to an unknown plant-working group), the tool would have been classified as ambiguous. Thus, the predictive model is quite consistent, as 13 experimental tools were correctly identified, and one was classified as unknown, so there was not any wrong classification.

**Table 3:** Predicted group membership through quadratic discriminant classification of the experimental tools. All the experimental tools are correctly classified (more than 55% of the samples classified in the correct group), except tool 10, which provides ambiguous results.

Predicted group membership									
Tool n°	Type of plant	Domestic ripe cereals		Wild cultivated semi-ripe cereals		Reeds		Grass	
		N	%	N	%	N	%	N	%
**1**	**Domestic ripe cereals**	16	84.2	3	15.8	0	0.0	0	0.0
**2**	**Domestic ripe cereals**	30	69.8	11	25.6	2	15.4	0	0.0
**3**	**Domestic ripe cereals**	20	83.3	4	16.7	0	0.0	0	0.0
**4**	**Domestic ripe cereals**	18	81.8	4	18.2	0	0.0	0	0.0
**5**	**Domestic ripe cereals**	11	61.1	5	27.8	2	28.6	0	0.0
**6**	**Domestic ripe cereals**	23	95.8	1	4.2	0	0.0	0	0.0
**7**	**Wild cultivated semi-ripe cereals**	4	20.0	15	75.0	1	6.3	0	0.0
**8**	**Wild cultivated semi-ripe cereals**	3	15.0	17	85.0	0	0.0	0	0.0
**9**	**Wild cultivated semi-ripe cereals**	7	33.3	13	61.9	0	0.0	1	2.9
**10**	**Wild cultivated semi-ripe cereals**	8	33.3	10	41.7	6	37.5	0	0.0
**11**	**Wild cultivated semi-ripe cereals**	2	9.5	19	90.5	0	0.0	0	0.0
**12**	**Reeds**	1	5.6	2	11.1	15	88.2	0	0.0
**13**	**Reeds**	1	4.8	1	4.8	18	90.0	1	4.2
**14**	**Grass**	0	0.0	0	0.0	0	0.0	15	100.0

Our predictive model allows a good rate of correct classification of gloss samples. However, there is a certain degree of confusion between ripe and semi-ripe cereal harvesting, as 18.5% of the samples of ripe harvesting are grouped as semi-ripe cereal cutting, and 22.9% of semi-ripe cutting are confused with ripe cereal cutting. Reed cutting can be confused with semi-ripe cereal cutting in 13.6% of cases. Thus, to ensure a correct classification of the archaeological tools, a high proportion of 3D image samples should be classified in one group. This high proportion is, at least, 55% of the samples, as suggested by the classification of experimental tools.

### Discriminating between different harvest heights

To discriminate between different harvest heights, four experimental sickles were used for several hours to cut two species of wheat (*Triticum dicoccum* and *Triticum spelta)* in Quinzanas (Asturias, Spain) either near the ground or under the spikelets [109]. Cultivation of the cereals in the replication experiment took place in a traditional way (e.g., without using herbicides), so many weeds were present in the fields at the time the stems were cut. For each experimental tool, the number of measured areas (650×500 μm) varies from 12 to 17, for a total of 111 subsamples of 200×200 μm (Table 4). Quadratic discriminant analysis was used to compare the 3D scans obtained from the archaeological assemblages that have been classified as cereal harvesting implements.

**Table 4:** The experimental program with the number of sampled areas and subareas for each tool used for testing usewear patterns produced when different heights of plants were cut. All the experiments were published in Mazzucco et al. 2022, Supplementary material 2.

Type	Tool	N°zones	N° subareas
**EXP**	Triticum spelta-high-E3	17	23
**EXP**	Triticum spelta-low-E2	13	39
**EXP**	Triticum dicoccum-high-E2	12	26
**EXP**	Triticum dicoccum-low-E1	15	23
	**Total**	57	111

Quadratic discriminant analysis running with SPSS software was used to compare the 3D scans obtained from the archaeological assemblages that have been classified as cereal harvesting implements in the first test. Two predictors that had been already successfully tested in Mazzucco et al. 2022 to discriminate between low- and high-height cutting experiments were used: *Smc* (inverse areal material ratio) and *Sxp* (peak extreme height). Significant mean differences (Wilks’ Lambda <0,001) were observed for both predictors and for the discriminant function. Using these parameters, 90% of the 3D samples were correctly classified (Table 5).

**Table 5:** Predicted group membership through quadratic discriminant classification of the experimental samples. 90.1% of original grouped cases were correctly classified.

		Predicted Group Membership		Total
		High	Low	
**N**	**High**	41	8	49
	**Low**	3	59	62
**%**	**High**	83.7	16.3	100
	**Low**	4.8	95.2	100

### Photographic recording

Macroscopic pictures have been made with a Hirox 3D Digital microscope and a digital SLR camera (Canon EOS 1300D) equipped with an EF-S 60 mm macro lens. Micrographs were taken with a digital SLR camera (Canon EOS 550D) attached to the Olympus microscope via an adapter (OM-Mount Photomicro Adapter L).

## Results

Results are presented together for sickle inserts from the West (WT) and East (ET) Mounds at Tappeh Sang-e Chakhmaq. This was done to highlight the similarities and the variability in farming technology and in patterns of cereals harvesting associated with the occupational levels at the two mounds. The studied sample is well preserved. The surfaces of the tools were slightly affected by post-depositional alterations (limited to fresh cone initiations scars, restricted areas of abrasion and long and deep random striations unrelated to use, which are easily distinguishable from usewear features). Thermal alterations are uncommon, affecting only 25 blades— 18 items from WT and 12 items from ET.

### Techno-morphological traits of the sickle elements

The most common lithic raw-material used to manufacture sickle elements coming from the two mounds was a fine-grained light brown chert (WT: n = 43; ET: n = 46; burnt pieces are not included here). There also were a few coarse-grained pieces of this type of chert (WT: n = 10; ET: n = 16). Other types include fine-grained brown (WT: n = 18; ET: n = 13), grey (WT: n = 16; ET: n = 9) and dark cherts (WT: n = 1), also with rare coarse-grained pieces (ET: n = 1 brown and 3 grey). This range of lithic raw-materials represents the local chert nodules commonly used at the site for the chipped stone production (see above). Most of the blades (WT: n = 102; ET: n = 97) and bladelets (WT: n = 4; ET: n = 3) were manufactured by pressure flaking on single-platform cores to produce very regular blade and bladelet blanks, with parallel and straight edges and ridges, small and smooth butts, and a trapezoidal (84%, i.e., WT: n = 88; ET: n = 84), triangular (10%, i.e., WT: n = 10; ET: n = 11) or plano-convexe cross-section (6%, i.e., WT: n = 8; ET: n = 5). Some thicker and irregular blades were produced by percussion. The blanks are mostly coming from the *plein debitage*, i.e., the central blades. There are only 5 lateral blades, i.e., 3 secondary crested blades (WT: n = 1; ET: n = 2) and 2 cortical blades (ET: n = 2).

**Fig 2:**
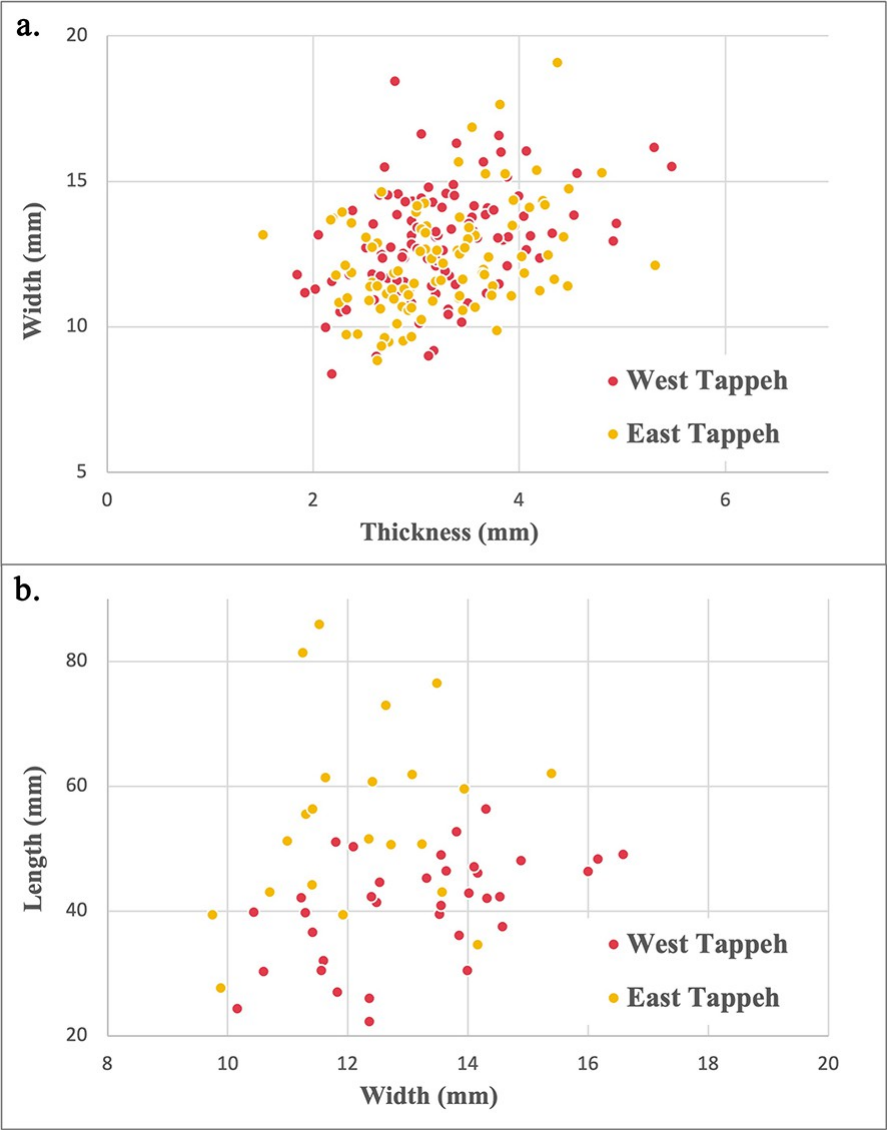
Scatter plots with sickle inserts measurements for each Mound. **a**. Width/Thickness. **b**. Width/Length.

The blanks are approximately 20–50mm long, 10–15 mm width and 2–5 mm thick for the WM (averages: 34.86x12.90x3.23 mm) and 20–80 mm long, 10–15 mm width and 2–5 mm thick for the EM (averages: 41.52x12.32x3.24 mm; Fig 2A). Thus, the metric analysis of blade/bladelet width (blanks less than 1 cm in wide are considered as bladelets) and thickness ranges suggests that there was some standardisation in sickle blade/bladelet production. The lengths of the whole preserved sickle inserts– i.e., without post-use breakage or retouch –ranges between 44.64 and 56.39 mm (average: 40.87 ± 8.46) for the 37 pieces coming from the West Mound and between 27.68 and 85.91 mm (average: 55 ± 15) for the 22 pieces coming from the East Mound. The sickle inserts tend to be longer in the East Tappeh assemblage (Fig 2B), with an average length/width ratio of 4.52 as against 3.11 for the West Mound.

Sickle elements are manufactured from blanks by a variety of retouching processes, which includes reduction of blank size by breakage (WT: n = 48; ET: n = 18), truncation (WT: n = 4; ET: n = 2) and/or backing (WT: n = 7; ET: n = 3) before use, and by the modification of the cutting edges (WT: n = 98/159; ET: n = 108/140). Typologically, both assemblages are composed of unretouched blades (WT: n = 33; ET: n = 19), blades with lateral retouch (WT: n = 48; ET: n = 54), denticulated blades (WT: n = 11; ET: n = 20), backed and denticulated blades (WT: n = 1; ET: n = 2), backed blades with lateral retouch (WT: n = 6; ET: n = 1), a truncated blade (ET = 1), truncated blades with denticulated (WT: n = 1) or lateral retouch (WT: n = 3; ET: n = 1). The morphology of the sickle blades corresponds to the type 5 defined for Northern Mesopotamia and Central Zagros in Late PPNB / Early PN [84]. Some sickle elements have been recycled as scrapers (WT = 2), as a drill (WT = 1) and as burins (ET = 2). Among the whole preserved sickle elements (WT: n = 37; ET: n = 22), proximal blade fragments are the most common (WT = 21; ET = 13), followed by the medial (WT = 8; ET = 6) and distal segments of the blanks (WT = 1; ET = 1). Complete blade blanks are rare (WT = 7; ET = 2).

### Cutting-edges characteristics and harvesting usewear traces

Using the high-power reflected-light microscope, 159 plant-harvesting used areas were identified among the 106 glossy blades from the WT and 140 plant-harvesting used areas among the 100 blades from the ET. Around half of them exhibit two active edges (WT = 53; ET = 40). Among the blades with only one cutting edge, the gloss appears more often on the right edge in the West Mound (WT = 41) than on the left one (WT = 12). The opposite is recorded during the occupation of East Mound, with 41 blades used on the left edge and 19 on the right edge. The cutting edges are mostly modified by a direct or indirect semi-abrupt to abrupt retouch (WT: n = 98/159, i.e., 62%; ET: n = 108/140, i.e., 77%). They are more rarely unretouched (WT: n = 61/159, i.e., 38%; ET: n = 32/140, i.e., 23%). In the sample from WT, 39% of the sickle inserts with visible gloss were retouched during the use (n = 98/159). In the ET sample, 28% were retouched during the use (n = 39/140). Apparently, they were retouched to resharpen the sickle edges.

**Fig 3:**
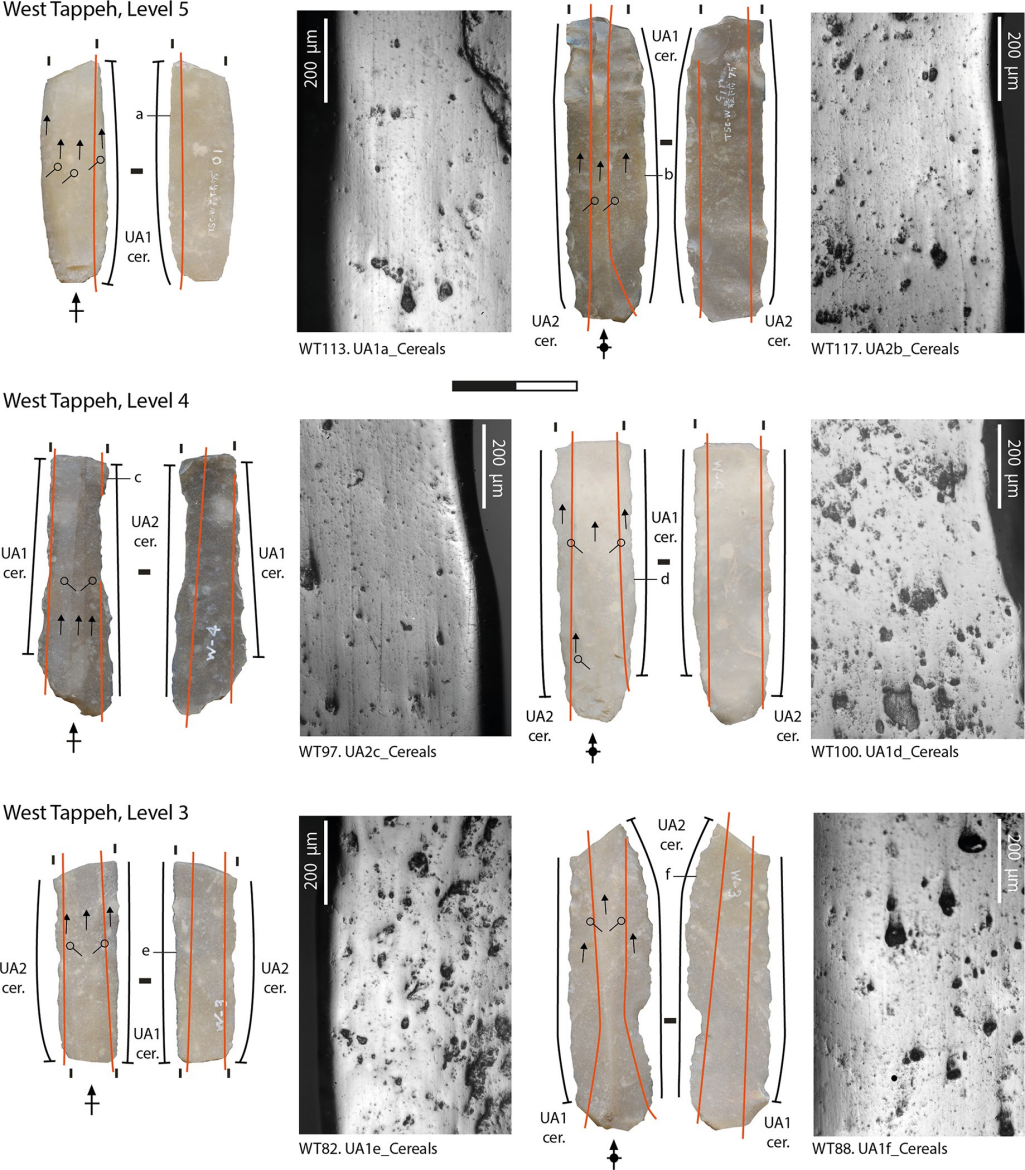
Archaeological usewear micropolish from cereal harvesting observed on the sickle inserts from the West Mound (levels 5–3).

**Fig 4:**
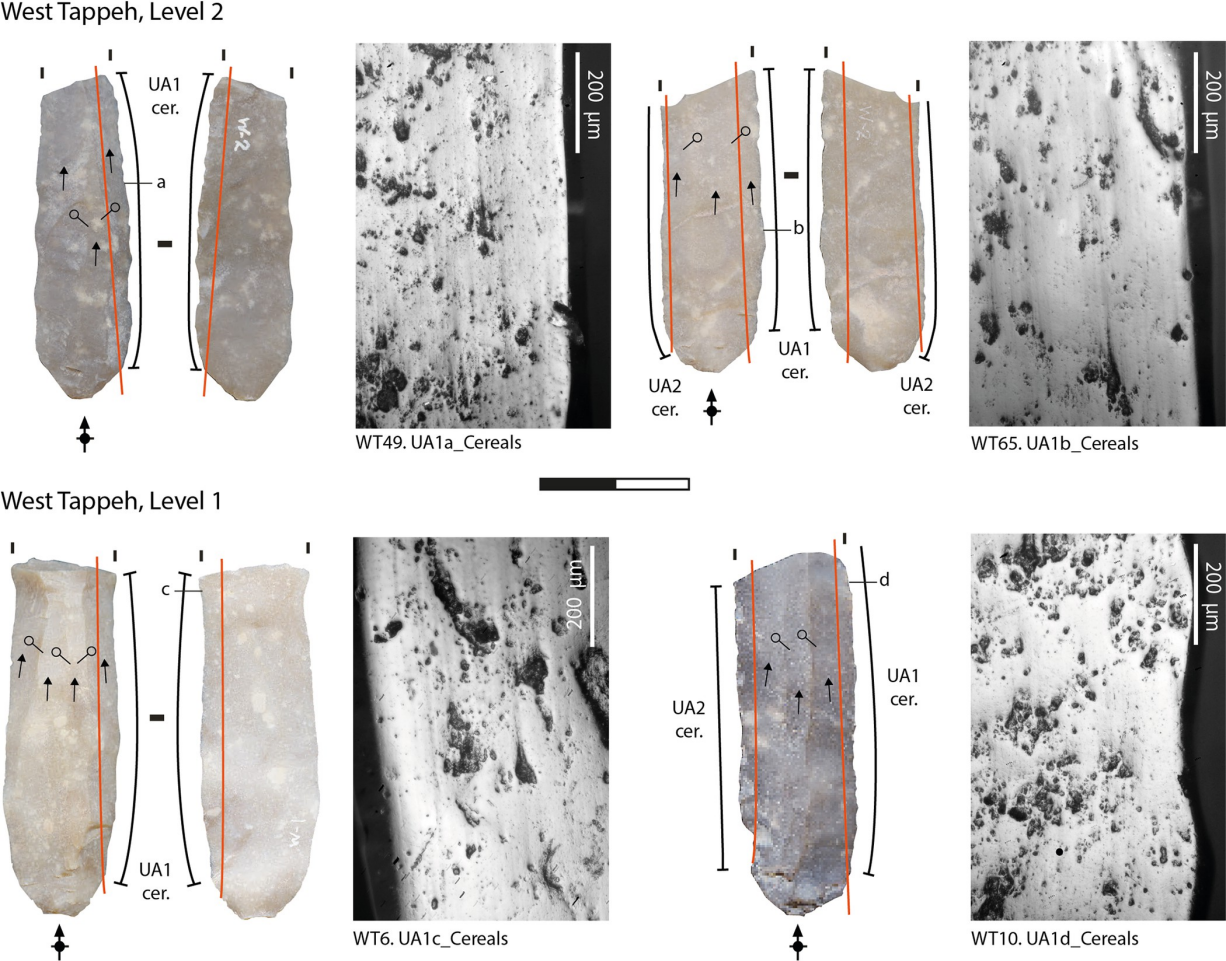
Archaeological usewear micropolish from cereal harvesting observed on the sickle inserts from the West Mound (levels 2–1).

**Fig 5:**
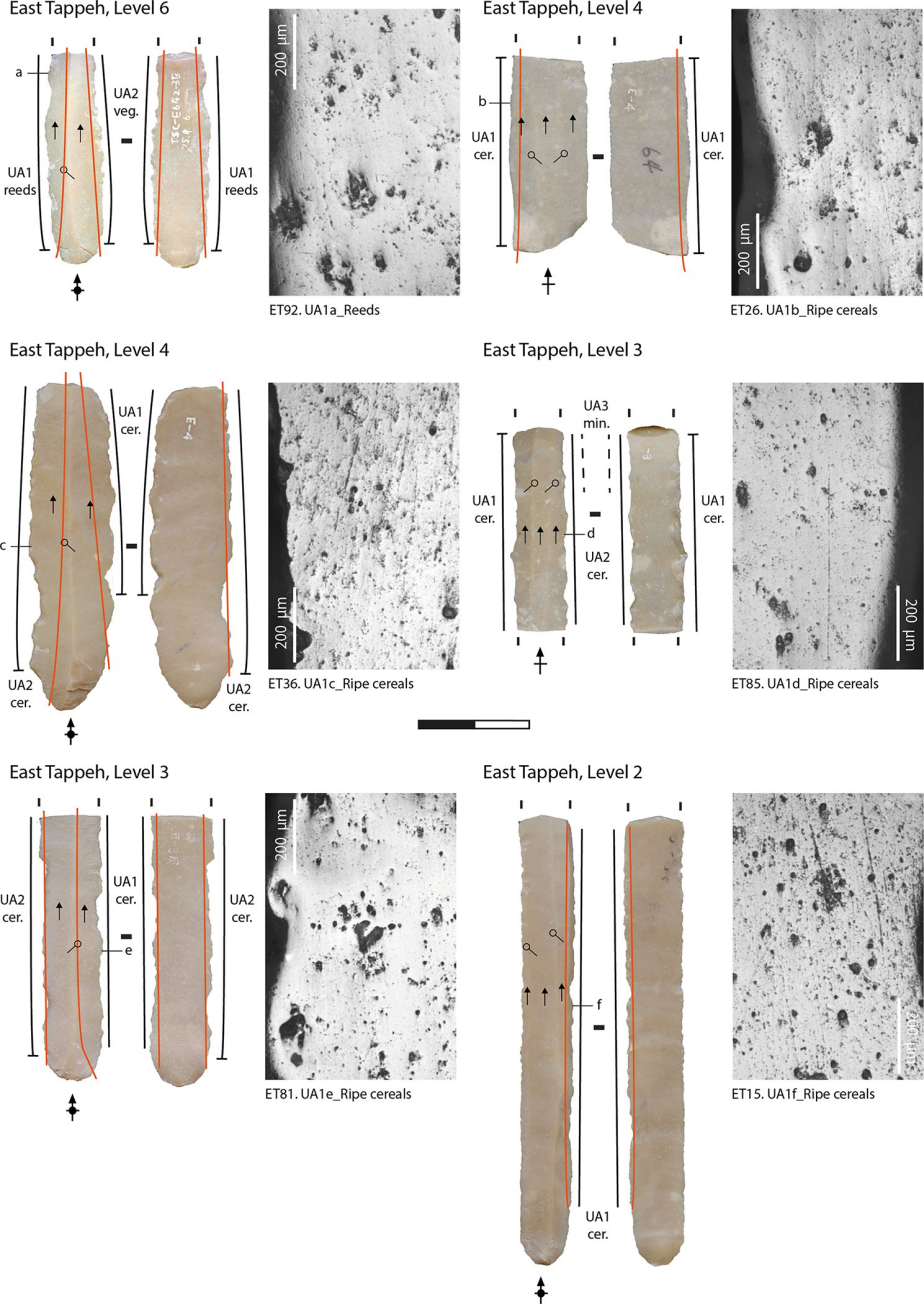
Archaeological usewear micropolish from cereal harvesting observed on the sickle inserts from the East Mound.

Most of the sickle inserts display a well-developed and very extensive band of polish along both the dorsal and ventral faces of their edges (at least up to 1 mm back from the edge of the blank), that exhibits the same general microscopic features that formed on the experimental tools used to harvest cereals (Figs 3–5) [101, 110, 111]. The micropolish is compact, smooth to slightly rough in texture, and associated with filled-in striations (i.e., long, narrow, and dark striations filled with siliceous material) and "comet-like" striations (linear comet-shaped pits), oriented parallel to the rounded cutting edge ([88, 110, 112–114]. The polish tends to be flat in topography and dull in appearance. The texture of the raw material may have played a role in the formation of the micropolish on the lower areas of the topography of the coarser chert blades, although the gloss is highly developed [111, 115]. Some variability is noticed among the whole assemblage in the amount of abrasion and the number of striations and comet-shaped pits along the cutting edges. This could be linked to several factors, such as cereals stem moisture and hardness [101] and occasional contact of the polished areas with abrasive mineral particles from the soil, which depends on the height of the cut and the characteristics of the soil [101, 111, 113, 116]. According to the identified usewear patterns, cereals probably were harvested in a ripe state of maturity, close to the ground at the base of the straw. This functional interpretation is supported by the analysis of sickle gloss texture that we applied to 25 blades (corresponding to 34 used areas) from the West Mound and 37 blades (corresponding to 47 different used areas) from the East Mound.

### Gloss microtexture analysis through confocal microscopy and metrology software

First, we will provide the results of texture analysis for all the 3D surfaces in global, considering the 920 sub-areas on sickle inserts from the West Mound and the 1119 sub-areas from the East Mound. We have grouped the archaeological tools into the four plant-cutting categories. The 3D image samples obtained from the same glossy edge can be classified into different groups. Firstly, there is a degree of uncertainty in our predictive model, as can be seen in the misclassification of experimental samples, mainly between ripe and semi-ripe cereal harvesting. Secondly, the same tool could have been used to harvest different types of plants successively. Although we have not tested it experimentally, we assume that when the same tool was used for cutting different types of plants, the resulting gloss would show mixed characteristics, so the tool would be classified as not assigned (less than 55% of the 3D images in any of the groups). However, our method is consistent enough to provide solid information about tendencies in harvesting habits in the past. For this, the statical tendencies must be considered.

**Fig 6:**
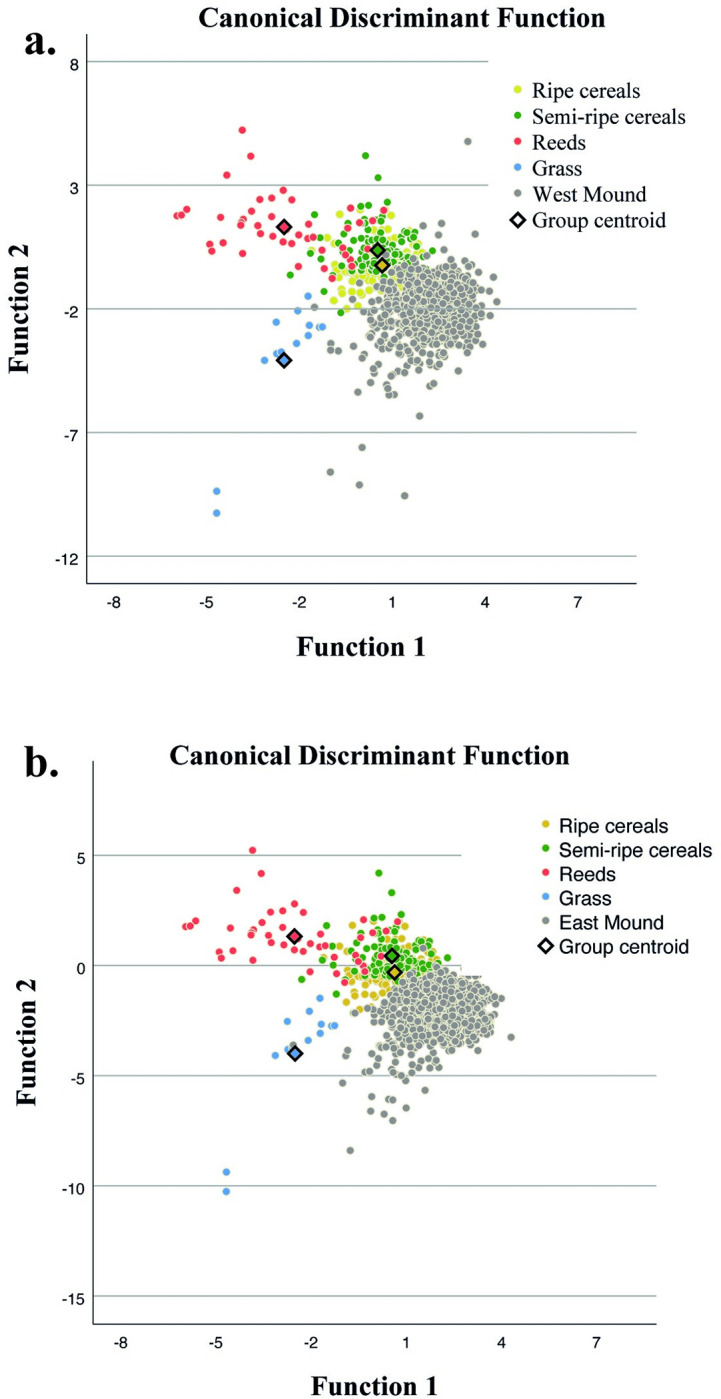
Results of the quadratic discriminant analysis of the experimental tools used for reaping domestic ripe cereals (yellow dots), wild-cultivated semi-ripe cereals (green dots), reeds (orange dots) and grass (blue dots) compared with the archaeological tools (grey dots) from the West (top) and the East (bottom) Mounds.

Results from glossed tools show that the most common type of gloss is similar to the pattern seen on experimental tools used for ripe cereal harvesting, with lower proportions of gloss that are similar to the patterns seen on experimental tools used for semi-ripe cereal harvesting, cutting reeds, and cutting grasses (Table 6; Fig 6). Some differences are observed between the abundance of different types of glossed tools from each tappeh. The dominance of ripe cereal harvesting gloss is clearer in the East Mound samples (70% of the classified surfaces), while in the West Mound, ripe cereal harvesting gloss was identified on 47% of the surfaces, whereas gloss from cutting reeds and semi-ripe cereals are more common than on the sickle inserts from the East Mound (Table 6).

**Table 6:** Classification of 3D samples of archaeological tools expressed as % by Mound with gloss similar to gloss pattern on experimental tools into the four plant cutting groups (domestic ripe cereals, wild-cultivated semi-ripe cereals, reeds, and grass).

Mound	N						%			
	Ripe	Semi-ripe		Reeds	Grass	Total	Ripe	Semi-ripe	Reeds	Grass
**West**	433		269	213	5	920	47.07	29.24	23.15	0.54
**East**	788		160	165	6	1119	70.42	14.30	14.75	0.54

There are some differences between the number of samples of 3D surfaces measured by tool, which varies between 16 and 40. Because of this, maybe the global result in biased by this disparity, as the tools with a larger number of surfaces weigh more in the global result than the tools with a smaller number of sampled 3D surfaces. To avoid potential bias, the weighted average was calculated (Table 7). These results are similar to the results in Table 6. Thus, the results of plant cutting groups did not differ significantly whether calculations are made considering the total number of samples or the average of the percentages of groups by tool (weighted average), both for the East (chi-square statistic: 10.1834; p-value: 0.01707) and the West mound (chi-square statistic: 13.4629; p-value: 0.003735).

**Table 7:** Classification of 3D samples of archaeological tools of both Mounds expressed as a weighted average (first, the percentage of the four groups of reaped plants per tool was calculated, and then, the average of the total percentages per mound was calculated).

Mound	%			
	Ripe	Semi-ripe	Reeds	Grass
**West**	45.23	31.53	22.62	0.62
**East**	69.15	15.90	14.38	0.57

Besides the assessment of the global results, considering all the 3D sampled surfaces, we calculated the results for each archaeological tool. Table 8 shows the classification of each 3D sampled area per tool. When we tested our predictive model classifying the experimental tools (Table 2), we saw that the threshold of 55% of 3D surfaces classified in one group was a good limit for the correct classification of a tool. Then, we used this threshold to classify the archaeological tools. When more than 55% of the 3D surfaces were classified in one group, the classification was considered as a correct one, while when the classification of any of the groups is lower than 55%, the tool was considered as not assigned. This threshold of 55% was also used in Ibáñez et al. 2016, to classify each tool for cutting one of the four types of plants (in that case, ripe cereal cereals, wild cultivated cereals, wild cereals in natural stands, and reeds). For the West Mound, 12 tools were used to harvest ripe cereals, 5 for semi-ripe cereals, and 2 for cutting reeds, while in the East Mound, 34 tools were used for reaping ripe cereals and 2 for cutting reeds (Table 9). On some tools (15 used areas in the WM and 11 used areas in the EM), the proportion of 3D surfaces in one of the groups did not reach 55%, so they were considered as ungrouped or not assigned. Most probably, these tools were used for cutting more than one type of plant, so the texture of the gloss shows mixed characteristics. In most of these tools, the addition of the 3D surfaces classified as ripe and semi-ripe cereals cutting is dominant compared with those identified as used for reed cutting, so these tools were most probably used for cutting cereals in a ripe/semi-ripe state. This happens in 11 of the 15 indeterminate tools from the West Mound and in 9 of the 11 tools from the East Mound.

**Table 8:** Number and percentage of cases where 3D sub-surface areas on each sickle insert in the samples from the two Mounds (WT, ET) were assigned to each of the four plant-cutting groups (domestic ripe cereals, wild-cultivated semi-ripe cereals, reeds and grass). When more than 55% of the 3D sub-surface areas on each insert were assigned to one of the four cutting groups, the tool was included in that group. When predicted membership in any of the four groups did not reach 55% of the 3D sub-surface areas, the tool was not assigned to any group.

Mound	Layer	Tool ID	N					%				
			Ripe	Semi-ripe	Reeds	Grass	Total	Ripe	Semi-ripe	Reeds	Grass	Predictive Group
WT	5	WT113	28	7	5	0	40	70.0	17.5	12.5	0.0	Ripe
WT	5	WT114	18	1	16	0	35	51.4	2.9	45.7	0.0	Not assigned
WT	5	WT115	18	5	5	0	28	64.3	17.9	17.9	0.0	Ripe
WT	5	WT116	2	17	0	1	20	10.0	85.0	0.0	5.0	Semi-ripe
WT	5	WT117 RG	29	4	0	0	33	87.9	12.1	0.0	0.0	Ripe
WT	5	WT117 LF	30	0	1	0	31	96.8	0.0	3.2	0.0	Ripe
WT	4	WT97 RG	14	8	4	1	27	51.9	29.6	14.8	3.7	Not assigned
WT	4	WT97 LF	19	9	0	0	28	67.9	32.1	0.0	0.0	Ripe
WT	4	WT100	3	10	0	0	13	23.1	76.9	0.0	0.0	Semi-ripe
WT	4	WT109 RG	7	11	5	0	23	30.4	47.8	21.7	0.0	Not assigned
WT	4	WT109 LF	8	15	2	0	25	32.0	60.0	8.0	0.0	Semi-ripe
WT	3	WT33 RG	7	5	11	0	23	30.4	21.7	47.8	0.0	Not assigned
WT	3	WT33 LF	4	9	7	0	20	20.0	45.0	35.0	0.0	Not assigned
WT	3	WT70	7	8	15	0	30	23.3	26.7	50.0	0.0	Not assigned
WT	3	WT76	3	16	13	0	32	9.4	50.0	40.6	0.0	Not assigned
WT	3	WT85 RG	5	9	1	0	15	33.3	60.0	6.7	0.0	Semi-ripe
WT	3	WT85 LF	9	23	1	0	33	27.3	69.7	3.0	0.0	Semi-ripe
WT	3	WT90 RG	7	9	8	0	24	29.2	37.5	33.3	0.0	Not assigned
WT	3	WT90 LF	8	9	25	0	42	19.0	21.4	59.5	0.0	Reeds
WT	2	WT35 RG	20	8	5	1	34	58.8	23.5	14.7	2.9	Ripe
WT	2	WT35 LF	17	5	3	0	25	68.0	20.0	12.0	0.0	Ripe
WT	2	WT36	11	2	19	0	32	34.4	6.3	59.4	0.0	Reeds
WT	2	WT39	19	6	5	0	30	63.3	20.0	16.7	0.0	Ripe
WT	2	WT44	9	4	7	0	20	45.0	20.0	35.0	0.0	Not assigned
WT	2	WT61	10	13	10	0	33	30.3	39.4	30.3	0.0	Not assigned
WT	2	WT65	11	2	6	0	19	57.9	10.5	31.6	0.0	Ripe
WT	1	WT5	7	6	6	1	20	35.0	30.0	30.0	5.0	Not assigned
WT	1	WT6	6	4	10	0	20	30.0	20.0	50.0	0.0	Not assigned
WT	1	WT8	3	9	14	0	26	11.5	34.6	53.8	0.0	Not assigned
WT	1	WT9 RG	30	6	1	0	37	81.1	16.2	2.7	0.0	Ripe
WT	1	WT9 LF	25	4	0	0	29	86.2	13.8	0.0	0.0	Ripe
WT	1	WT10 RG	11	9	2	1	23	47.8	39.1	8.7	4.3	Not assigned
WT	1	WT10 LF	18	6	2	0	26	69.2	23.1	7.7	0.0	Ripe
WT	1	WT24	10	10	4	0	24	41.7	41.7	16.7	0.0	Not assigned
*Total sub-samples*			*433*	*269*	*213*	*5*	*920*					
ET	6	ET88	18	1	1	0	20	90.0	5.0	5.0	0.0	Ripe
ET	6	ET89	16	0	10	0	26	61.5	0.0	38.5	0.0	Ripe
ET	6	ET90	8	1	11	0	20	40.0	5.0	55.0	0.0	Reeds
ET	6	ET91	16	0	15	0	31	51.6	0.0	48.4	0.0	Not assigned
ET	6	ET92	7	3	13	0	23	30.4	13.0	56.5	0.0	Reeds
ET	6	ET93 LF	16	3	2	0	21	76.2	14.3	9.5	0.0	Ripe
ET	6	ET93 RG	14	7	8	0	29	48.3	24.1	27.6	0.0	Not assigned
ET	6	ET95	23	1	1	0	25	92.0	4.0	4.0	0.0	Ripe
ET	6	ET96	15	0	1	0	16	93.8	0.0	6.3	0.0	Ripe
ET	6	ET99 LF	35	0	1	0	36	97.2	0.0	2.8	0.0	Ripe
ET	6	ET99 RG	17	3	0	0	20	85.0	15.0	0.0	0.0	Ripe
ET	6	ET100	8	7	0	0	15	53.3	46.7	0.0	0.0	Not assigned
ET	5	ET50	20	3	4	0	27	74.1	11.1	14.8	0.0	Ripe
ET	5	ET58	15	4	2	0	21	71.4	19.0	9.5	0.0	Ripe
ET	5	ET59	24	4	0	0	28	85.7	14.3	0.0	0.0	Ripe
ET	5	ET61 LF	26	3	9	0	38	68.4	7.9	23.7	0.0	Ripe
ET	5	ET61 RG	27	1	5	0	33	81.8	3.0	15.2	0.0	Ripe
ET	5	ET63 RG	12	0	12	0	24	50.0	0.0	50.0	0.0	Not assigned
ET	5	ET63 LF	11	4	4	0	19	57.9	21.1	21.1	0.0	Ripe
ET	5	ET68	11	6	1	0	18	61,1	33,3	5.6	0.0	Ripe
ET	5	ET72	10	4	7	0	21	47.6	19.0	33.3	0.0	Not assigned
ET	5	ET77	15	4	1	0	20	75.0	20.0	5.0	0.0	Ripe
ET	5	ET78	23	3	4	0	30	76.7	10.0	13.3	0.0	Ripe
ET	5	ET79 LF	24	3	6	0	33	72.7	9.1	18.2	0.0	Ripe
ET	5	ET79 RG	21	0	0	0	21	100.0	0.0	0.0	0.0	Ripe
ET	4–5	ET42 RG	11	6	2	0	19	57.9	31.6	10.5	0.0	Ripe
ET	4–5	ET42 LF	25	3	2	0	30	83.3	10.0	6.7	0.0	Ripe
ET	4–5	ET43 RG	22	4	0	0	26	84.6	15.4	0.0	0.0	Ripe
ET	4–5	ET43 LF	14	3	0	0	17	82.4	17.6	0.0	0.0	Ripe
ET	4–5	ET46	15	1	0	1	17	88.2	5.9	0.0	5.9	Ripe
ET	4	ET26	22	5	2	0	29	75.9	17.2	6.9	0.0	Ripe
ET	4	ET27	6	8	3	1	18	33.3	44.4	16.7	5.6	Not assigned
ET	4	ET36 RG	14	2	0	0	16	87.5	12.5	0.0	0.0	Ripe
ET	4	ET36 LF	33	2	0	0	35	94.3	5.7	0.0	0.0	Ripe
ET	4	ET37 RG	18	1	1	0	20	90.0	5.0	5.0	0.0	Ripe
ET	4	ET37 LF	11	4	1	0	16	68.8	25.0	6.3	0.0	Ripe
ET	3	ET80	9	8	1	0	18	50.0	44.4	5.6	0.0	Not assigned
ET	3	ET81	13	2	7	0	22	59.1	9.1	31.8	0.0	Ripe
ET	3	ET82	11	5	8	0	24	45.8	20.8	33.3	0.0	Not assigned
ET	3	ET83	21	1	1	0	23	91.3	4.3	4.3	0.0	Ripe
ET	3	ET84	16	4	2	0	22	72.7	18.2	9.1	0.0	Ripe
ET	3	ET85 LF	26	0	0	0	26	100.0	0.0	0.0	0.0	Ripe
ET	3	ET85 RG	26	1	1	0	28	92.9	3.6	3.6	0.0	Ripe
ET	3	ET86	3	11	7	0	21	14.3	52.4	33.3	0.0	Not assigned
ET	2	ET14	5	9	3	0	17	29.4	52.9	17.6	0.0	Not assigned
ET	2	ET15	31	2	1	0	34	91.2	5.9	2.9	0.0	Ripe
ET	2–3	ET19	4	13	5	4	26	15.4	50.0	19.2	15.4	Not assigned
*Total sub-samples*			788	160	165	6	1119					

**Table 9:** Summary of the classification of archaeological tools by Mound. Numbers represent individual used areas on sickle blades. When more than 55% of the samples from each archaeological tool were classified in one group, the tool was considered as belonging to that group. When predicted membership classification in any of the four groups did not reach 55% of the samples, the tool was not assigned to a group.

Mound	N					%			
	Ripe	Semi-ripe	Reeds	Not assigned	Total	Ripe	Semi-ripe	Reeds	Not assigned
**West**	10	5	2	15	34	35.29	14.71	5.88	44.12
**East**	31	0	2	11	47	72.34	0.00	4.26	23.40

**Fig 7:**
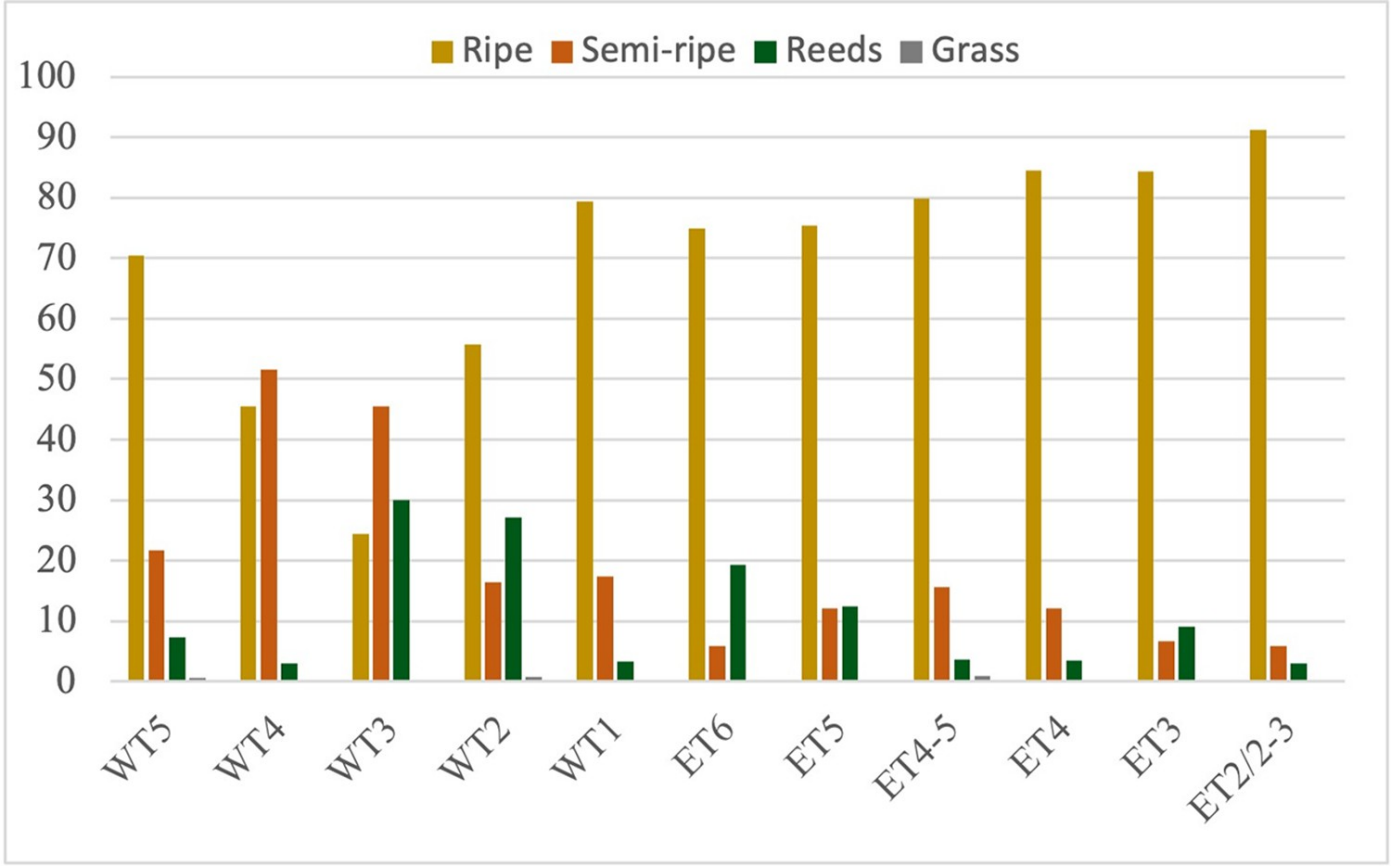
Sample frequency according to the classification of the 3D sub-surface areas into the four plant cutting groups by level for West and East Mounds.

By considering only the sickle inserts assigned to one of the four groups from each occupation level (threshold of 55%), organised by level, the evolution through time in the characteristics of the cut plants harvested by the inhabitants of both mounds over time can be examined in both mounds (Table 10; Fig 7). As not all the tools had the same quantity of surface samples, we pondered the results of the classification of the samples in base 10. For the West Tappeh mound occupations, harvesting ripe plants is the most common practice during the time of oldest (level 5) and most recent occupation levels (1 and 2). Semi-ripe plant harvesting is the most common practice during the time of the middle occupation levels (3 and 4). These fluctuations suggest some changes in the characteristics of the cereals harvested through time in the West Mound. Possibly cereals that needed to be harvested in a greener state were introduced into the site during the time of the level 4 and level 3 occupations in the West Mound. Ripe cereal reaping is the most common practice throughout the entire occupation sequence of the Eastern Mound (levels 6–2), whereas semi-ripe harvesting seems to be less common.

**Table 10:** Proportion of the 3D sub-surface areas into the four plant cutting groups by level for West and East Mounds, considering only the samples of the tools classified in one of the four groups (threshold of 55%).

Mound	Layer	N				%			
		Ripe	Semi-ripe	Reeds	Grass	Ripe	Semi-ripe	Reeds	Grass
**West**	**WT5**	107	33	11	1	70,39	21,71	7,24	0,66
** **	**WT4**	30	34	2	0	45,45	51,52	3,03	0,00
** **	**WT3**	22	41	27	0	24,44	45,56	30,00	0,00
** **	**WT2**	78	23	38	1	55,71	16,43	27,14	0,71
** **	**WT1**	73	16	3	0	79,35	17,39	3,26	0,00
**East**	**ET6**	155	12	40	0	74,88	5,80	19,32	0,00
** **	**ET5**	217	35	36	0	75,35	12,15	12,50	0,00
** **	**ET4-5**	87	17	4	1	79,82	15,60	3,67	0,92
** **	**ET4**	98	14	4	0	84,48	12,07	3,45	0,00
** **	**ET3**	102	8	11	0	84,30	6,61	9,09	0,00
** **	**ET2/2-3**	31	2	1	0	91,18	5,88	2,94	0,00

The images that were classified as being used for cereal harvesting were also tested to see if it was possible to discriminate between sickle blades used for a low- or high-height cutting on the plants. Only tools with more of 55% of the subzones classed as ripe, semi-ripe or ripe+semi-ripe were selected. Tested sickle blades are: 20 tools corresponding to 27 used areas for the West Mound and 34 tools corresponding to 43 active edges for the East Mound (Table 11).

**Table 11:** Selected tools and number of measured subareas for each mound (N°).

Mound	Layer	Tool ID	N° subareas	Mound	Layer	Tool ID	N° subareas
WT	5	WT113	40	ET	6	ET88	20
WT	5	WT115	28	ET	6	ET89	34
WT	5	WT116	20	ET	6	ET93 LF	21
WT	5	WT117 RG	33	ET	6	ET93 RG	29
WT	5	WT117 LF	31	ET	6	ET95	25
WT	4	WT97 RG	27	ET	6	ET96	16
WT	4	WT97 LF	28	ET	6	ET99 LF	36
WT	4	WT100	15	ET	6	ET99 RG	20
WT	4	WT109 RG	24	ET	6	ET100	15
WT	4	WT109 LF	25	ET	5	ET50	27
WT	3	WT33 LF	21	ET	5	ET58	21
WT	3	WT76	32	ET	5	ET59	29
WT	3	WT85 RG	15	ET	5	ET61 LF	38
WT	3	WT85 LF	33	ET	5	ET61 RG	33
WT	3	WT90 RG	24	ET	5	ET63 LF	19
WT	2	WT35 RG	34	ET	5	ET68	18
WT	2	WT35 LF	25	ET	5	ET72	22
WT	2	WT39	30	ET	5	ET77	21
WT	2	WT44	20	ET	5	ET78	30
WT	2	WT61	34	ET	5	ET79 LF	33
WT	2	WT65	22	ET	5	ET79 RG	21
WT	1	WT5	20	ET	4–5	ET42 RG	19
WT	1	WT9 RG	37	ET	4–5	ET42 LF	30
WT	1	WT9 LF	29	ET	4–5	ET43 RG	27
WT	1	WT10 RG	23	ET	4–5	ET43 LF	18
WT	1	WT10 LF	26	ET	4–5	ET46	17
WT	1	WT24	24	ET	4	ET26	29
		** **	** **	ET	4	ET27	18
		** **	** **	ET	4	ET36 RG	16
		** **	** **	ET	4	ET36 LF	35
		** **	** **	ET	4	ET37 RG	20
		** **	** **	ET	4	ET37 LF	16
		** **	** **	ET	3	ET80	19
		** **	** **	ET	3	ET81	22
		** **	** **	ET	3	ET82	24
		** **	** **	ET	3	ET83	23
		** **	** **	ET	3	ET84	22
		** **	** **	ET	3	ET85 LF	26
		** **	** **	ET	3	ET85 RG	28
		** **	** **	ET	3	ET86	22
		** **	** **	ET	2–3	ET19	27
				ET	2	ET14	17
				ET	2	ET15	34
*Total sub-samples*			*720*	*Total sub-samples*			*1037*

The results of the classification based on the results of the low and high height cutting experiments indicate that 98% of the 1757 analysed subareas—720 3D sub-samples for West Mound and 1037 3D sub-samples for East Mound —are classified as having been used for reaping at low height (Table 12).

**Table 12:** Predicted group membership of the 3D subsamples measured on the sickle blades through quadratic discriminant classification of the two groups (low and high height cutting).

Predicted groups						
Mound	N			%		
	Low	High	Total	Low	High	Total
**West**	696	24	720	93.7	6.3	100,00
**East**	1028	9	1037	99.1	0.9	100,00
**Total**	1724	33	1757	98.12	1.9	100,00

### Hafting mode

The parallel band of harvesting gloss along the utilised edges and the longitudinal orientation of the striations on all of the sickle inserts in the WT and ET samples indicate that they were hafted parallel to the long axis of the handle. The dimensions of the whole preserved implements— average of 40.87 mm for West Tappeh and 55 mm for East Tappeh—suggests that several sickle blades were inserted in the shaft to form the cutting edge of the sickles. Sickle inserts were subject to blank modification by breakage, truncation, and backing before use (see above). Rare brown/black residues have been observed on a few blades, opposite from the cutting edge, showing some use of adhesive to secure the sickle inserts in the haft.

The distribution of the polished area is generally straight along the cutting edges (Figs 3–5). In some cases, the usewear does not cover one or both extremities–i.e., proximal or distal ends—of the blades (WT = 35 used areas; ET = 73 used areas). This distribution pattern matches what would be expected on the inserts in the decorated bone sickle handles in level 5 of the Eastern Tappeh (no handles were found in West Tappeh) [90, 92]. The groove of the sickle handle is straight, with a slight curvature at its ends. According to the distribution of gloss on the sickle blades, this type of hafting could have been used during the entire time that the site was inhabited.

The main difference in the harvesting technology between the two mounds is related to the length of the sickle blade inserts, which are slightly longer in the East Tappeh. One of the longer ones— between 8 to 9 cm—could have been hafted in a sickle handle without any other inserts since the dimensions of the groove in the preserved shafts, which does not exceed 10 cm (a single flint blade insert was found mounted in one preserved haft [90]). The distribution of gloss on the longer and well-preserved sickle inserts does not extend to their proximal and distal ends (Fig 5. ET15).

### Other uses and recycling

Evidence for the use of sickle inserts for tasks other than harvesting is rare in both the Tappeh West and Tappeh East samples. Only 13% of the blades from the West Mound and 9% from the East Mound have other types of usewear traces. Recycling of sickle blades for another activity was identified on 13 blades in the West Mound (there were 15 reused areas on 15 edges with harvesting traces) and 6 blades in the East Mound (corresponding to 9 reused areas on 7 edges with harvesting traces).

**Fig 8:**
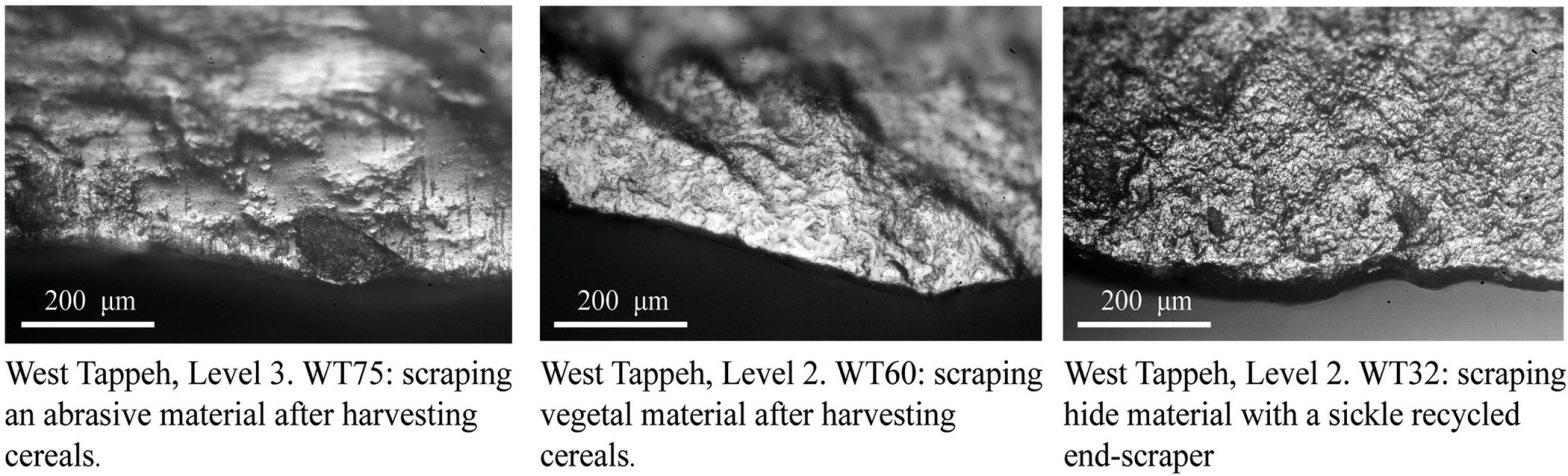
Examples of other usewear traces observed on some recycled sickle blades from West Tappeh.

In the West Mound, the other activities identified were scraping wood (3 used areas), scraping siliceous plants (2 used areas), scraping vegetal material (3 used areas; Fig 8. WT60), scraping hide (3 used areas: Fig 8. WT32), drilling an unidentified mineral (1 used area) and scraping indeterminate materials (10 used areas; Fig 8. WT25), In the East Tappeh, the represented activities were scraping bone (3 used areas), scraping mineral (2 used areas including one with red residues), cutting hide (2 used areas), scraping hide (1 used area), scraping plants (1 used area) and scraping indeterminate soft abrasive material (4 used areas). That means that sometimes sickle inserts that had been used for harvesting were recycled and reused for other activities.

## Discussion

### Harvesting practices at Tappeh Sang-e Chakhmaq

Our results show that when the Neolithic community arrived at Tappeh Sang-e Chakhmaq and established the West Mound settlement (WT) in the late eighth millennium BCE, cereals were harvested when they were fully ripened. Archaeobotanical analysis revealed that most of the harvested cereals in the WT levels were one- and two-grained einkorn (*T*. *monococcum*) and a tetraploid hulled wheat (*T*. *turgidum* or *T*. *timophevii*) as the main cereals taxa since the earliest levels, while barley is poorly represented [74, 99]. Free-threshing wheat (*T*. *aestivum*) was incorporated into the subsistence economy of the East Mound (ET) Neolithic village, while einkorn is absent, suggesting a change in the crop assemblage through time [99]. Discriminant analysis of gloss texture allowed us to clearly classify most of the sickle inserts in the WT and ET samples as tools for harvesting ripe cereals (10 tools corresponding to 10 used areas for the West Mound and 26 tools corresponding to 31 active edges for the East Mound). However, there is some variation in harvesting practices between the two mounds. The cutting of semi-ripe cereals is recorded in the western mound (29% of the harvesting tools in which the state of ripeness was identified), while only ripe cereals were reaped in the eastern mound. In a previous study [27], we observed the progressive evolution towards the harvesting of riper cereals in the Levant. In the Middle Euphrates, the first sickle elements with traces of cutting ripe cereals appeared during the PPNA, though they were still lower in number when compared sickle elements with evidence of cutting green and semi-ripe cereals. During the Early PPNB, the proportion of ripe cereal harvesting increased though it was not dominant until the Middle PPNB at Tell Halula (Syria) when domesticated cereals were identified. During the Late PPNB of Tell Halula (dated to around 7,000 cal BCE), evidence for harvesting ripe cereals was found on 55.5% of the sickle elements [27]. Consistent with this trend, our data indicate that in samples from the lower (older) occupation levels of the Western Mound, there is evidence that not all the cereals were cut when they had reached a fully mature state. The harvesting of fully ripe cereals is evident in more recent times in our samples from level 2 of the West Mound and in all occupation levels of the East Mound. No sickle inserts with traces from harvesting semi-ripe plants were identified in these younger levels. Usewear patterns on sickle inserts in our samples that may have been used to cut reeds were not common (N = 2 in the WT and N = 2 in the ET). The proportion of 3D surfaces on other inserts in our samples that matched reed-cutting (or grass-cutting) patterns on experimental tools never reached more than 55%. However, some tools have been used to cut different types of plants affecting the textural characteristics of the micropolish. Archaeobotanical analyses showed that wild plants, including sedges (*Cyperaceae*), were gathered at Tappeh Sang-e Chakhmaq [74, 99], and the use of reeds as building material in houses has been documented in both mounds (i.e., marks of reed stalks thrust into the top of walls suggesting their use for the roof) [90]. Usewear traces suggest that harvesting was close to the ground, and that straw was collected along with the grain. This “low-cutting” procedure facilitated the transport of cereals in bundles from the field to the processing area. It is described in ethnographic accounts along with some of the ways that straw could be used for other tasks [117]. None of the inserts in our samples had microwear traces from use in threshing sledges [118–121]; therefore, it seems that other techniques were used for processing the crops. Alternative methods for separating base spikelets from straw could be either hitting with a wooden tool (i.e., a flail) or trampling with animals on the threshing floor, allowing the break of the straw into small pieces as well. Chaff has been used as a temper for pottery [122] and for building materials (pisé walls and mudbricks—A. Tsuneki, Personal communication). The low-cutting of cereal stems is a well-known prehistoric agricultural practice, documented at several Neolithic sites in the Levant and in Cyprus [123, 124]. For example, the presence of low-growing weed species at Tell Qarassa North [24] or Dja’de el-Mughara [125] suggests that cereals have been harvested close to the ground.

### The origins of agriculture in NE Iran

Besides original data on harvesting technology in Tappeh Sang-e Chakhmaq, our study can provide some points of reflection on the agricultural process of cereal cultivation in northeastern Iran. Current interpretations of the Neolithic transition suggest the dispersal of farming practices and technologies from the Eastern Fertile Crescent (EFC) north-eastward across Iran and beyond into western Central Asia [67]. For now, chronological, biogeographical, archaeobotanical, and genetic data indicate that domesticated ungulates (sheep and goats) and crops (einkorn and barley) identified in the southeastern Caspian Sea area, northeastern Iran and southwestern Turkmenistan were probably introduced from the EFC, rather than locally domesticated [58, 62, 67, 126]. Most significantly, aDNA analysis of Neolithic EFC human remains have provided an insight into the population dynamics that occurred during the transition to farming in Iran, identifying the Zagros as the cradle of an eastern expansion of human farming communities probably travelling with southwestern Asian domestic animals and plants [127]. A recent study [76] proposed two major pathways for the introduction of the first domesticated plants and animals east to the Zagros Mountains by cultural or demic diffusions. A northern pathway would extend from southeastern Turkey towards the southeastern Caspian Sea, the Alborz Mountains and southern regions. A southern pathway would have crossed through the Zagros and Taurus fringes towards the northern edge of the Central Desert, across the Zanjan and Qazvin plains. The documented obsidian circulation networks support the hypothesis of the Central Zagros valleys as a possible communication route, from which obsidian originating from eastern Anatolian sources reached the northeast region of Iran during the late eighth millennium [68, 128]. The existence of such widespread networks of contact must have played a fundamental role in the possible adoption and diffusion of the Neolithic way of life from Zagros eastward by enhancing the circulation of materials, ideas, and innovations between communities.

The data on harvesting practices in Tappeh Sang-e Chakhmaq fits a community of pioneer farmers who settled down in the area carrying with them both domestic cereals and knowledge of advanced techniques of cereal cultivation as well. Interestingly, comparing the degree of ripeness of harvested cereals recorded at Tappeh Sang-e Chakhmaq with the Northern Levantine context, the data of the Western Mound show a slightly more advanced degree of maturity than the one documented at the Late PPNB levels of Tell Halula (55% *vs* 59%) where cereal domestication is well attested [27]. The fluctuation in the degree of ripeness of harvested cereals along the first four levels of occupation of the Western Mound suggests that cereals needing harvesting before full maturity were introduced into the village. From the topmost layer I of the Western Mound and all along the occupation of the Eastern Mound, ripe harvesting is clearly dominant, showing a well-established cultivation strategy of fully mature cereal.

The sickle inserts in our samples were made from flint blades which were inserted in parallel in straight handles. In the East Mound, handles made of bone and decorated with animal carvings were recovered [90, 92]. Our interest is whether the sickle manufacturing technologies of Tappeh Sang-e Chakhmaq during the late eighth-millennium cal BCE could indicate a relationship with the Fertile Crescent, in particular its eastern fringe, the region that is thought to have played an important role in the Neolithisation of the northeastern Iranian plateau [67, 76]. A complete crescent bone haft discovered at Zawi Chemi Shanidar was clearly designed to hold microlithic inserts, with a central groove ranging from 1–3 mm in width [129]. A remarkable example of an intact straight bone haft was found as a grave good in level B1 of the cemetery in Shanidar Cave, tentatively dated to ca. 10,500 cal BCE [129, 130]. The handle, perforated at one end, contains a single flint blade fixed in parallel into the opposite extremity of the handle with a lavish application of bitumen. In Bestansur, inhabited from 7700 and 7100 cal. BCE, one fragment of bone haft with bitumen was recovered [131]. Our preliminary functional analysis of the glossy blades indicates the parallel insertion of the blades into straight hafts. A four-element sickle was found in J-I.7 at Jarmo [132], dated to ca. 7 000 cal BCE [133]. There is no trace of the handle, but the general shape of the sickle, revealed by the distribution of the three flint elements and the bitumen, is curved with parallel inserts [132]. As there are some sites in the Zagros where straight sickles were used earlier than the occupation of Tappeh Sang-e Chakhmaq, it is possible to suggest that the sickles originate from the Zagros area. However, this is still hypothetical, given the lack of a systematic analysis of the sickle characteristics in the two potential areas of origin (the Zagros region or SE Turkey) of the farmers who settle down in NE Iran at the end of the 8^th^ millennium BCE.

Regarding the characteristics of sickles in neighbouring regions coeval to Chakhmaq, several straight sickle hafts with short grooves (< 10 cm), decorated with animal heads [134] found in Tepe Sialk I, dated around 6,000–5,500 cal BCE [135] are very similar to the sickle hafts discovered at Tappeh Sang-e Chakhmaq East [90]. Similar types of hook-shaped bone sickle hafts are also present in the Djeitun-period sites in Southern Turkmenistan [67]. At the end of the seventh millennium cal BCE, the presence of a straight sickle decorated with carved animal heads in the Zagros (Sialk), the Iranian plateau (Chakhmaq) and the Djeitun culture indicate the existence of a shared tradition in harvesting technology. The identification of straight sickles in the Djeitun Culture in Turkmenistan, like those found in Chakhmaq, would fit well with the hypothesis of its Iranian origins, suggesting that this region could have been an intermediate step in the expansion of farming towards the East.

## Conclusion

The analysis of sickle elements recovered in the West and East Mounds at Tappeh Sang-e Chakhmaq, Iran, shows that almost all these tools were used for cereal harvesting, although a small number were used for cutting reeds. The comparison of wear traces on archaeological and experimental sickle inserts suggests that cereals were usually harvested in a ripe state and were cut near the ground. This reconstructed pattern of cereal cultivation is supported by archaeobotanical data. However, changes in harvesting practices can be observed between the first four levels of occupation of the Western Mound, where harvesting semi-ripe cereals is important, and the more recent levels of occupation of the mounds, where harvesting of fully ripe cereals was carried out. This shift could indicate an *in-situ* evolution towards a better-established agricultural system that included harvesting fully ripe crops, which would have resulted in higher yields. Regarding the origins of the harvesting technology, harvesting tools were knives. This kind of tool is documented in some previous sites of the central Zagros, so this could be the origin of the harvesting tool. However, our knowledge about harvesting tools in SE Turkey, the other potential origin of the Chakhmaq farming communities, and even about the Zagros region is so meagre that this question is still open.

## Supporting information

S1 Checklist (DOCX)
